# Exercise preconditioning mitigates brain injury after cerebral ischemia‐reperfusion injury in rats by restraining TIMP1

**DOI:** 10.1002/iid3.70008

**Published:** 2024-10-04

**Authors:** Xiangbo Meng, Hui Yang, Feifeng Chen, Baohua Li, Yan Wu, Rong Wang

**Affiliations:** ^1^ Department of Rehabilitation Medicine The Affiliated Hospital of Hangzhou Normal University Hangzhou 310000 Zhejiang Province China; ^2^ Department of Neurology Hangzhou First People's Hospital Hangzhou 310006 Zhejiang Province China; ^3^ Department of Rehabilitation Medicine Hangzhou First People's Hospital Hangzhou 310006 Zhejiang Province China; ^4^ Department of Radiology Hangzhou First People's Hospital Hangzhou 310006 Zhejiang Province China

**Keywords:** brain injury, cerebral ischemia‐reperfusion injury, exercise preconditioning, TIMP1

## Abstract

**Background:**

Cerebral ischemic disease is a common cerebrovascular disease, especially ischemic stroke. Exercise has protective functions on brain tissues following cerebral ischemia‐reperfusion injury (CIRI), but its preventive effects and mechanisms in CIRI remain unclear. We aimed to investigate the effects and mechanisms of exercise preconditioning on CIRI.

**Methods:**

The middle cerebral artery occlusion (MCAO) operation was prepared to establish CIRI rats. All rats were randomized into the MCAO, exercise (exercise preconditioning plus MCAO operation), vector (exercise preconditioning, MCAO operation plus intraventricular injection of empty vector), and tissue inhibitor of metalloprotease 1 overexpression (OE‐TIMP1, exercise preconditioning, MCAO operation plus intraventricular injection of OE‐TIMP1) groups.

**Results:**

The results indicated that exercise preconditioning suppressed approximately 66.67% of neurological deficit scores and 73.79% of TIMP1 mRNA expression in MCAO rats, which were partially offset by OE‐TIMP1. The protective effects of exercise against neuron death status and cerebral infarction size in MCAO rats were reversed by OE‐TIMP1. It also confirmed that exercise weakened apoptosis and oxidative stress damage, with notable increases of B‐cell lymphoma‐2, superoxide dismutase, and glutathione peroxidase production, and evident decreases of BCL2‐associated X, caspase 3, and malondialdehyde in MCAO rats, while these effects were partially reversed by OE‐TIMP1. Additionally, the inhibitory effects of exercise on the protein levels of TIMP1, hypoxia‐inducible factor‐alpha, vascular endothelial growth factor receptor 2, vascular endothelial growth factor, and neurogenic locus notch homolog protein 1 in MCAO rats were partially reversed by OE‐TIMP1.

**Conclusion:**

Altogether, exercise preconditioning had protective effects on CIRI by restraining TIMP1, which provided new therapeutic strategies for preventing CIRI.

## Graphical abstract

We found that exercise preconditioning had protective effects on CIRI by restraining TIMP1, which provides new targets and therapeutic strategies for the prevention of CIRI.

## 1 INTRODUCTION

Cerebral ischemia is a leading cause of disability and death worldwide, posing a serious threat to human health and quality of life.^1^ Ischemic encephalopathy is the most common cerebrovascular disease, especially ischemic stroke (IS), accounting for 60%‐80% of all cerebrovascular diseases.^2^ IS happens when intracerebral vessels become obstructed.^3^ This interrupts the cerebral supply of oxygen and nutritive substances, which results in the permanent infarction of brain tissues and further causes numerous acute neurologic deficits.^4^ A study including data on the incidence and mortality of IS in China has revealed that from 1990 to 2019, the age‐standardized incidence rate of IS increased by 35.0%, and the age‐standardized mortality rate increased by 3.0%.^5^ Furthermore, globally, there is an increase in IS incidence with increasing age, particularly in women aged 50 to 69 years or older.^6^ In recent years, despite huge efforts that have been made, the treatment of ischaemic disease remains challenging clinically.^7^ For example, treatment methods such as embolectomy and thrombolysis have certain risks, which may lead to ischemia‐reperfusion injury (IRI) and increase the risk of bleeding.^8^ Cerebral IRI (CIRI) is a type of injury caused by severe inflammatory reactions and oxidative stress after the cerebral tissues in ischemia and hypoxia suddenly recover the blood supply.^9^ Due to the physiological and behavioral similarities between rats and humans, rats are considered excellent animal models for studying human diseases. Therefore, it is of great significance to actively seek therapeutic methods to prevent CIRI using rat models.

Tissue inhibitor of metalloproteinase‐1 (TIMP1) is a member of the TIMP family, and an experiment from human specimen sections has indicated that TIMP1 expression in the infarcted brain tissue was higher than that in the normal brain tissue.^10^ Another report has proved that the concentration of TIMP1 in the rat penumbral zone enhances prominently after 24 h of CIRI and that TIMP1 plays a crucial role in the inflammatory response in the acute phase of CIRI.^11^ Therefore, we speculate that inhibition of TIMP1 expression may exhibit a significant role in the neuroprotective process following CIRI.

Exercise preconditioning is an intervention method to conduct a certain amount of exercise before the occurrence of cerebral ischemia to generate ischemic tolerance.^12^ Related consensus such as cardiovascular and cerebrovascular diseases lists exercise as a primary prevention and recommends a certain amount of daily exercise to prevent the occurrence of diseases.^13^, ^14^ On the other hand, exercise preconditioning can also improve ischemia tolerance, increase neurovascular integrity, promote nerve regeneration, and reduce the inflammatory response after CIRI, as confirmed by a large number of experiments.^15^, ^16^ In addition, compared with other preconditioning methods, exercise preconditioning has many advantages, such as convenient implementation, safer, wider audience, and greater acceptable.^17^ Some scholars have suggested that exercise combined with drug therapy can regulate the level of TIMP1, thereby ameliorating diabetes and lung injury.^18^, ^19^ However, it is still unknown whether exercise can improve CIRI by modulating TIMP1.

In our previous study, we used RNA sequencing to detect that the gene with the most significant expression difference in middle cerebral artery occlusion (MCAO) rats with exercise intervention was TIMP1. We also found that exercise preconditioning could regulate the hypoxia‐inducible factor (HIF) pathway to participate in the improvement of exercise on CIRI. However, the specific mechanisms were still uncertain. Therefore, in this experiment, we established the MCAO model in rats undergoing exercise preconditioning, and studied the effects of exercise preconditioning on TIMP1 expression, exploring the mechanisms of exercise preconditioning on brain protection.

## 2 MATERIALS AND METHODS

### 2.1 Animals

Male Sprague‐Dawley rats weighing 250–300 g and with a specific pathogen‐free class were introduced from Shanghai Jihui Laboratory Animal Care Co., Ltd. and reared at Zhejiang Eyong Pharmaceutical Research and Development Center. All rats were allowed to adapt to the environment (25 ± 2°C, the humidity of 55 ± 5%, and 12‐h of circulating light) for 7 days. Subsequently, the rats were randomized into the MCAO, exercise, vector, and TIMP1 overexpression (OE‐TIMP1) groups (*n* = 20 per group).

### 2.2 Exercise intervention

All rats in the exercise, vector, and OE‐TIMP1 groups underwent adaptive training for 3 days before formal exercise training, that is, training for 20 min per day on an electric treadmill with a 0° incline and a speed of 10 m/min. Formal training began after the completion of adaptive training, that is, on the electric treadmill with a 0° incline and a speed of 15 m/min, training for 30 min per day, 6 days a week for 3 weeks.^20^ An electric shock zone was set up at the back of the rat‐running platform. Rats that refused to run would fall into the electric shock zone and receive an electric shock with an intensity of 1.5 mA. During this period, the MCAO group of rats underwent 3 weeks of free exercise without any exercise intervention.

### 2.3 Lentivirus treatment

The TIMP1 overexpression lentiviral vectors and empty lentiviral vectors were bought from YouBio (China). 2 h after completion of the exercise intervention, the rats were sedated deeply with 3% isoflurane and then maintained by 2% isoflurane.^20^ Anesthetized rats lay prone on an operating table with their heads fixed on a stereotaxic device, and the scalp was open to expose the skull. According to the preliminary experiments, the 2‐point injection was finally determined and the injection dose was 5 μL OE‐TIMP1 vector or negative control (1 × 10^9^ TU/mL) for each rat.^21^ Point A: 1.0–2.0 mm in front of the anterior fontanelle, 3.0 mm to the left of the centerline, and 3.0 mm in injection depth; Point B: 0.5–1.5 mm posterior to anterior fontanelle, 3.5 mm to the left of the centerline, and injection depth of 3.5 mm.^22^, ^23^, ^24^ The injection speed was adjusted to 0.5 μL/min. Animals of the MCAO, and exercise groups were injected with the same amount of photosphate buffered saline intraventricularly.

### 2.4 Animal models of MCAO

After 1 week of lentivirus treatment, the MCAO rats models were conducted as follows^25^: the anesthetized rats were put on a temperature‐controlled warming pad to keep a body temperature of 37 ± 0.5°C throughout the procedure. The right common carotid artery (CCA) was carefully exposed and separated, and the CCA was ligated at the more proximal side through a right paramedian incision. The external carotid artery (ECA) was also ligated. Ischemia was produced by advancing the tip of a rounded 3–0 suture into the internal carotid artery through the ECA. After 90 min of ischemia, nylon sutures were slowly pulled out to restore blood flow and reperfusion was formed. Then, the wound was sewn carefully. Following surgery, rats that underwent MCAO surgery were administrated with Penicillin G (60,000 U/rat) and 20 mg/kg tramadol (50 mg/mL) subcutaneously to prevent infection and provide analgesia.^26^, ^27^ A video camera was used to monitor the appearance, activity and appetite of the rats. The mortality of each group was recorded 48 h after MCAO surgery.

### 2.5 Assessment of neurologic deficit scores

After 48 h of MCAO surgery. The neurologic deficit score of each rat was determined using modified Longa scores.^28^ The scoring criteria were as follows: 0 point for no deficits; 1 point for being unable to extend the contralateral forelimb and the mild neurologic deficit; 2 points for moderate neurological impairment and contralateral steering during crawling; 3 points for severe neurological impairment and lateral inclination during crawling; 4 points for unable to walk spontaneously coupled with the loss of consciousness; 5 points for death. To ensure the reliability of the results, rats with abnormal neurologic deficit scores in each group were eliminated, and then 9 rats from each group were selected randomly for following experiments.

### 2.6 Obtaining tissue specimens

After the complement of neurological function measurement, rats were euthanasia by inhaling excessive CO_2_. Three rats from every group were selected for 2,3,5‐triphenyl tetrazolium chloride (TTC) staining. The remaining brain tissues were dissected and the cerebral cortex tissues were separated. The cerebral cortex tissues for Nissl staining, and terminal deoxynucleotidyl‐transferase‐mediated dUTP nick end labeling (TUNEL) staining were fixed in 4% paraformaldehyde. After dehydration, the tissues were embedded in paraffin and cut into sections. The remaining cerebral cortex tissues were exploited for enzyme‐linked immunosorbent assay (ELISA), quantitative real‐time polymerase chain reaction (qRT‐PCR), and Western blot tests.

### 2.7 QRT‐PCR

As previously,^29^ the isolation of total RNA from the cerebral cortex tissues was carried out with the use of the EZ‐10 Total RNA Mini‐Preps Kit before being converted to complementary DNA. Then, the complementary DNA was amplified with SYBR Green qPCR kits (11201ES08, Yeasen Biotech, China) under a PCR system (LightCycler® 96, Roche, USA). The fold change was determined by 2^‐ΔΔCt^, with β‐actin serving as the internal reference. Primers used in the study were listed in Table 1.

**Table 1:** Primer sequence information for qRT‐PCR.

Gene	Forward Primer	Reverse Primer
Rat TIMP1	AGCTTTCTGCAACTCGGACC	TCGAGACCCCAAGGTATTGC
Rat β‐actin	AAGGCCAACCGTGAAAAGAT	GCTCGAAGTCTAGGGCACA

### 2.8 Nissl staining

To estimate the status of neuronal cell death in the cerebral cortex sections of each group, Nissl staining was conducted.^30^ The cerebral cortex sections were dewaxed and hydrated, and then stained with the Nissl staining solution (C0117, Beyotime, China) for 8 min. After that, the stained sections were dehydrated and transparent, followed by sealing with neutral balsam. Finally, the stained results were captured using an optical microscope (Eclipse Ci‐L, Nikon, Japan).

### 2.9 Determination of cerebral infarction

The area of cerebral infarction was measured using the TTC staining.^31^ Brain tissues were frozen and then cut into acquired serial coronal Section (2 mm thick). Next, the sections were immersed in TTC solution (T8170, Solarbio, China) for 30 min at 37°C in the darkness. After washing, the sections were bathed in 4% paraformaldehyde overnight. Lastly, the infarction size was calculated using ImageJ software (version 1.52, National Institutes of Health) with the formula: infarction size (%) = (sum of white ischemic areas from brain slices)/(sum of brain slice areas) × 100%.

### 2.10 TUNEL staining

The apoptosis of the cerebral cortex sections was evaluated by TUNEL staining as previously.^32^ Briefly, the samples were dewaxed in xylene and processed in gradually reduced concentrations of ethanol. Afterward, the slides underwent proteinase K for 30 min. Following rinsing, the sections were subjected to TUNEL solution (C1090, Beyotime, China) in darkness (37°C, 1 h). After that, an Anti‐fade Mounting Medium with 4′,6‐diamidino‐2‐phenylindole (ab104139, Abcam, UK) was added. In the end, the results were observed and photographed by a fluorescence microscope (IX70, Olympus, Japan).

### 2.11 ELISA assay

To examine oxidative stress‐associated factors in cerebral cortex tissues, Jiancheng (China) supplied superoxide dismutase (SOD) kits (A001‐3), malondialdehyde (MDA) kits (A003‐1), and glutathione peroxidase (GSH‐Px) kits (A005‐1). In brief, the cerebral cortex samples were treated with lysate followed by homogenization. Next, the homogenized cerebral cortex samples were centrifuged to obtain the supernatant. The levels of the aforementioned markers were examined as per the operation manual.^33^

### 2.12 Western blot assay

As previously, Western blot assay was applied to evaluate the protein levels in the cerebral cortex.^34^ To extract total protein from the cerebral cortex samples, radio‐immunoprecipitation assay buffer was utilized. Following quantification with bicinchoninic acid kits, the protein underwent denaturation and electrophoresis. Next, the protein was loaded onto polyvinylidene fluoride membranes, which were then treated with 5% bovine serum albumin at 37°C for 60 min. The strips were then incubated with primary antibodies at 4°C overnight, before being immersed in anti‐rabbit IgG H&L (HRP) antibodies (1:6000, 7074, CST, USA) or anti‐mouse IgG H&L (HRP) antibodies (1:6000, 7076, CST, USA) at 37°C for 1 h. Enhanced chemiluminescence luminescence reagents were utilized to test specific protein signals. The membrane was exposed and photographed under a gel imaging system (610020‐9Q, Clinx, China). The primary antibodies were presented in Table 2.

**Table 2:** Primary antibody information for Western blot assay.

Antibody	manufactor	Article No	dilution rate
Bcl‐2 Antibody	Affinity	AF6139	1:1000
Bax Antibody	Affinity	AF0120	1:1000
caspase 3 Antibody	Affinity	AF6311	1:1000
TIMP1 Antibody	Affinity	AF7007	1:1000
HIF‐α Antibody	Affinity	BF8002	1:1000
VEGF Antibody	Affinity	DF7470	1:1000
VEGFR2 Antibody	Affinity	AF6281	1:1000
Notch1 Antibody	Abcan	ab52627	1:1000
β‐actin Antibody	Proteintech	81115‐1‐RR	1:10000
GAPDH Antibody	Proteintech	10494‐1‐AP	1:10000

### 2.13 Statistical analysis

All experiments were conducted at least 3 times to ensure the reliability and statistical significance of the results. Statistical analysis was performed using statistical product and service solutions software (20.0, IBM, USA). The data of the study were presented as mean ± standard deviation. One‐way analysis of variance with Turkey tests were utilized for multiple comparisons. Dunnett's T3 test was applied when the data distribution was normal but the variance was not uniform. If the data distribution was not normal, the Kruskal‐Wallis H test was employed for the nonparametric analysis. A *P‐*value < 0.05 was considered significant.

## 3 RESULTS

### 3.1 The suppression of exercise on the brain dysfunction and the mRNA level of TIMP1 in MCAO rats was neutralized by OE‐TIMP1

**Figure 1:**
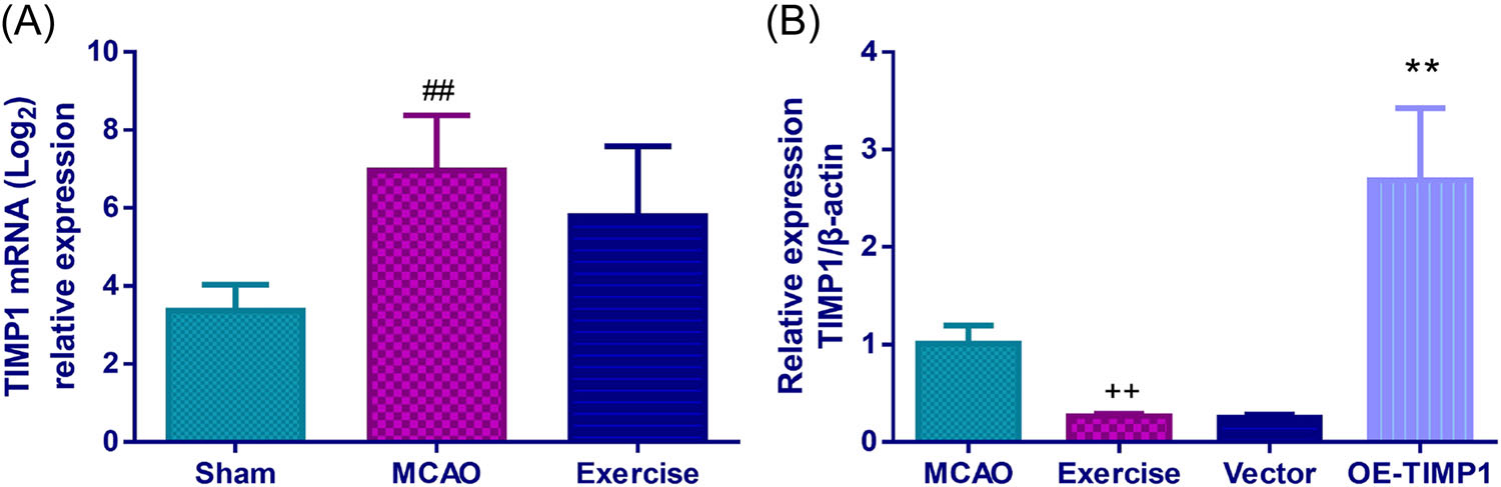
Exercise alleviated neurological deficits in MCAO rats by restraining TIMP1. (A) Relative expression of TIMP1 mRNA in MCAO rats pretreated with or without exercise according to RNA sequencing, *n* = 5, ^##^ *p* < .01 versus Sham. (B) The effects of exercise and TIMP1 overexpression on the mRNA level of TIMP1 of the cerebral cortex tissues in MCAO rats were examined by qRT‐PCR. ^++^ *p* < .01 versus MCAO; **p < .01 versus Vector. Data were analyzed using One‐way ANOVA with Turkey tests. *n* = 3. Results were presented as mean ± SD in all figures. Note: MCAO, middle cerebral artery occlusion; TIMP1, tissue inhibitor of matrix metalloprotease 1; qRT‐PCR, real‐time quantitative PCR; ANOVA, analysis of variance; SD, standard deviation.

**Figure 2:**
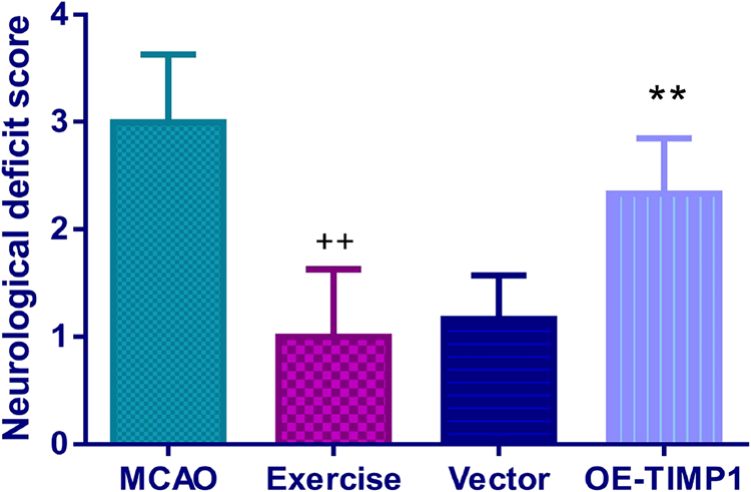
The effects of exercise and TIMP1 overexpression on the neurological deficit scores in MCAO rats. The neurological deficit score of each rat was assessed 48 h after MCAO. ^++^ *p* < .01 versus MCAO; **p < .01 versusVector. Data were analyzed using One‐way ANOVA with Turkey tests. *n* = 6. Results were presented as mean ± SD.

In our previous study, RNA sequencing results showed that the mRNA expression of TIMP1 in the MCAO rats was higher than in the sham rats (*p* < .01), while exercise intervention restrained the mRNA level of TIMP1 in MCAO rats (Figure 1A). By conducting further qRT‐PCR assay, exercise treatment was found to repress the mRNA expression of TIMP1 in MCAO rats (Figure 1B, *p* < .01). In contrast, it showed a reverse trend in the OE‐TIMP1 group (Figure 1B, *p* < .01). The mortality rates for rats in the MCAO, exercise, vector, and OE‐TIMP1 groups were 30%, 10%, 10% and 20%, respectively. As displayed in Figure 2, exercise effectively weakened the neurological deficit scores in MCAO rats, which was basically eliminated by OE‐TIMP1 (*p* < .01).

### 3.2 The protective effects of exercise against the neuron death status in the cerebral cortex tissues were counteracted by OE‐TIMP1

**Figure 3:**
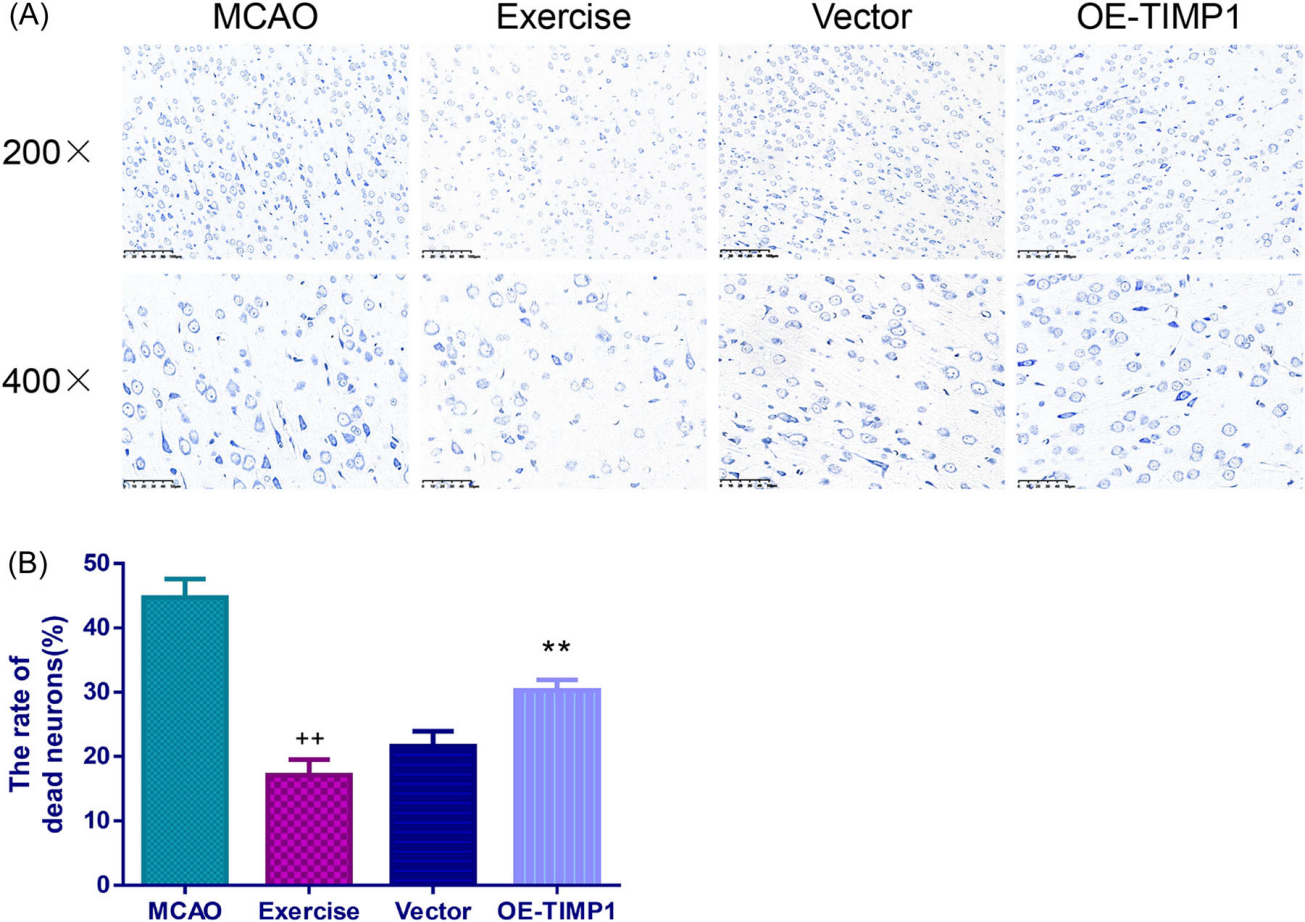
Exercise reduced cerebral cortex neuronal death in MCAO rats by inhibiting TIMP1. After performing MCAO surgery, the extent of neuronal death in the cerebral cortex of each rat was detected through Nissl staining. (A) Representative images of Nissl staining in the cerebral cortex. Magnification: 200×, scale bar: 100 μm, and magnification: 400×, scale bar: 50 μm. (B) Histograms of the rate of dead neurons obtained via Nissl staining. ^++^ *p* < .01 versus MCAO; **p < .01 versus Vector. Data were analyzed using One‐way ANOVA with Turkey tests. *n* = 3.

To estimate the neuron death status in the cerebral cortex sections of each group, Nissl staining was performed. The results showed that MCAO triggered a large number of neuron death (Figure 3, *p* < .01). However, exercise was found to alleviate the neuron death of MCAO rats (*p* < .01). Of note, the inhibitory effect of exercise on neuron death was partially reversed by OE‐TIMP1 (*p* < .01).

### 3.3 The effects of exercise and OE‐TIMP1 on the cerebral infarction size in MCAO rats

**Figure 4:**
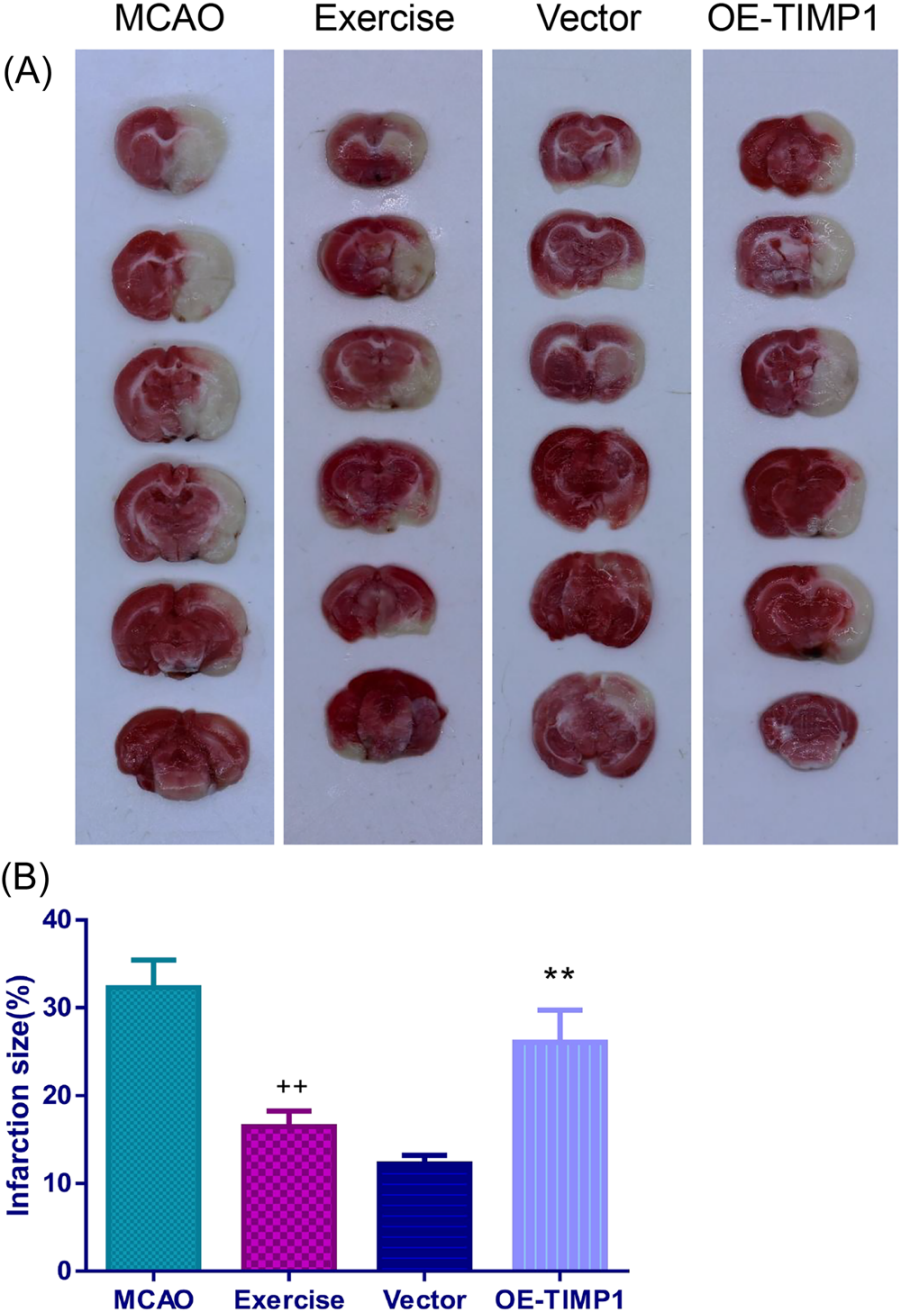
Exercise decreased cerebral infarct size in MCAO rats via weakening TIMP1. Following MCAO surgery, the extent of cerebral infarction from each group was evaluated using TTC staining. (A) Representative images of TTC staining. (B) Histograms of the infarction size obtained via TTC staining. ^++^ *p* < .01 versus MCAO; **p < .01 versusVector. Data were analyzed using One‐way ANOVA with Turkey tests. *n* = 3. TTC, Triphenyl tetrazolium chloride.

The TTC staining analysis revealed that the exercise group had a smaller cerebral infarction size compared to the MCAO group (Figure 4, *p* < .01). However, it was observed that OE‐TIMP1 largely abrogated the ability of exercise to alleviate MCAO‐mediated cerebral infarction size (*p* < .01).

### 3.4 The effects of exercise and OE‐TIMP1 on apoptosis of the cerebral cortex tissues in MCAO rats

**Figure 5:**
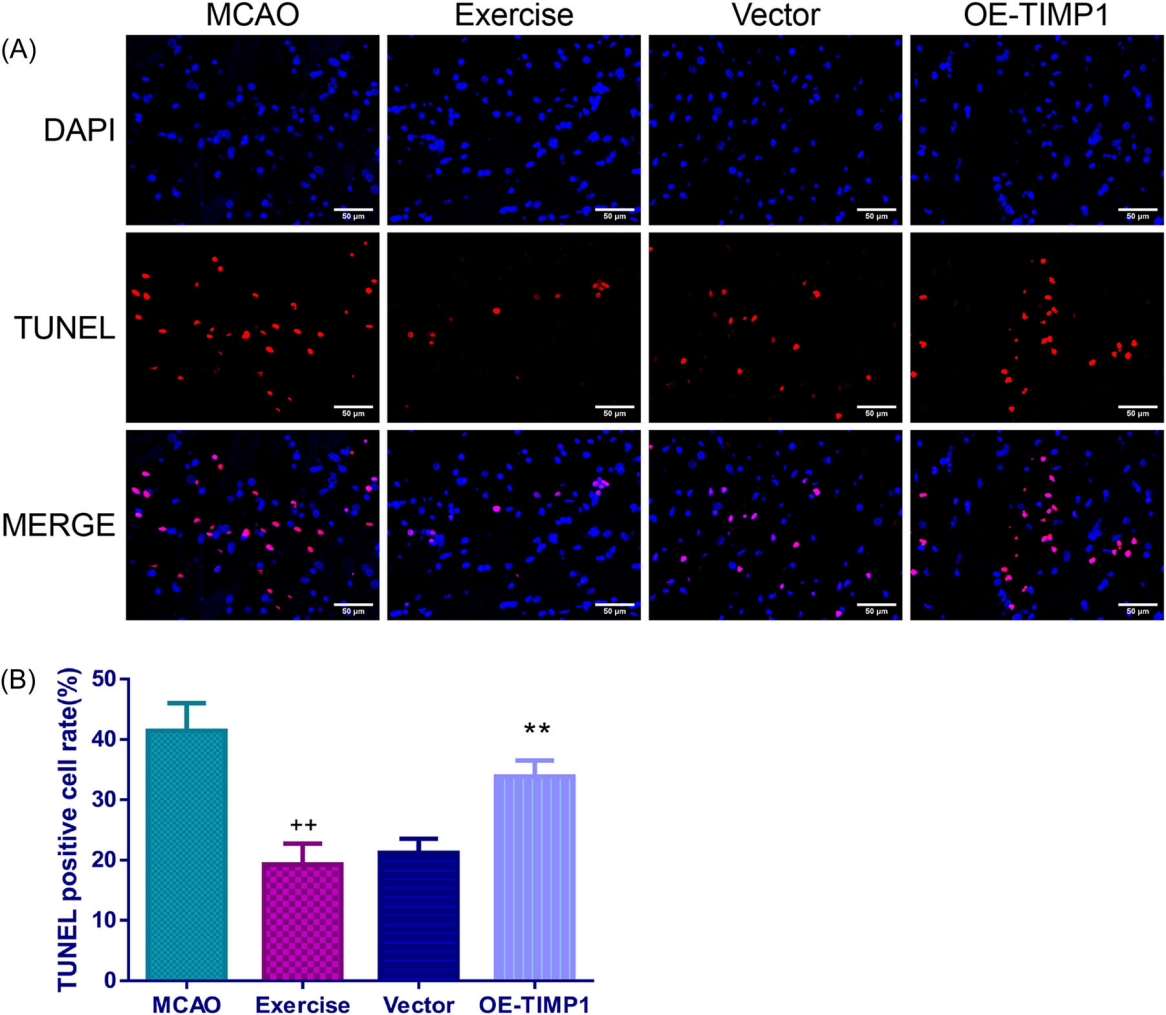
Exercise diminished the apoptosis of the cerebral cortex in MCAO rats by suppressing TIMP1. Upon conducting MCAO surgery, TUNEL assay was used to test apoptosis in rat cerebral cortex tissues. (A) Representative images of TUNEL staining in the cerebral cortex. Magnification: 200×, scale bar: 50 μm. (B) Histograms of the TUNEL positive cell rate obtained via TUNEL assay. ^++^ *p* < .01 versus MCAO; **p < .01 versus Vector. Data were analyzed using One‐way ANOVA with Turkey tests. *n* = 3. TUNEL, TdT‐mediated dUTP nick end labeling.

**Figure 6:**
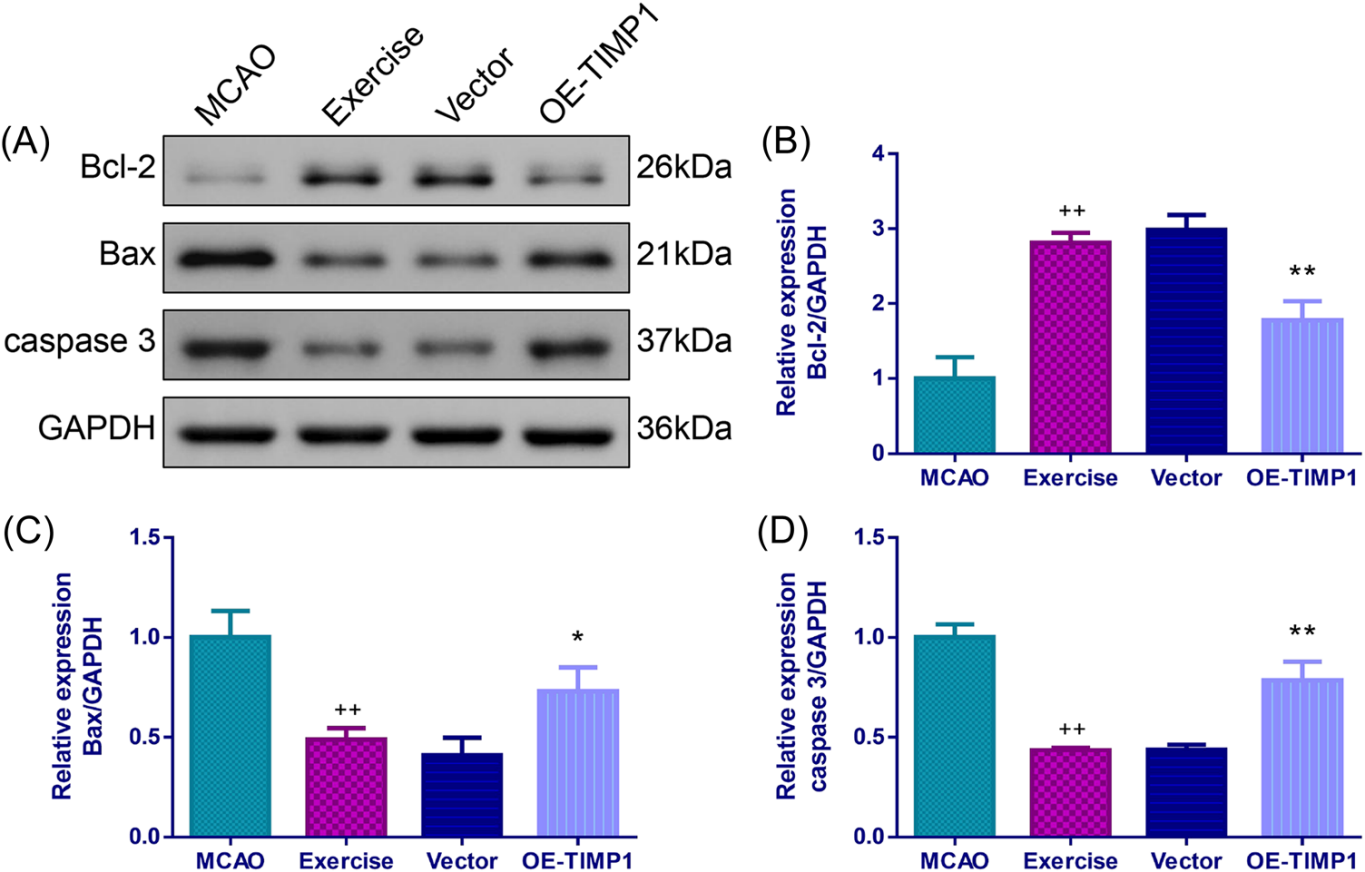
Exercise elevated Bcl‐2 expression but blocked Bax and caspase3 expressions in MCAO rats via impeding TIMP1. Following MCAO, the protein expressions of Bcl‐2, Bax, and caspase 3 in rat cerebral cortex were determined by Western blot assay. (A) Representative images for Western blot assay of Bcl‐2, Bax and caspase 3 levels in the cerebral cortex. Quantifications of Bcl‐2 (B), Bax (C) and caspase3 (D) levels in the cerebral cortex. ^++^ *p* < .01 versus MCAO; *p < .05, **p < .01 versus Vector. Data were analyzed using One‐way ANOVA with Turkey tests. *n* = 3. Bcl‐2, B‐cell lymphoma‐2; Bax, BCL2‐Associated X.

The TUNEL assay indicated that exercise preconditioning significantly suppressed the TUNEL positive cell rate (Figure 5, *p* < .01). Nevertheless, exercise preconditioning‐mediated reduction in apoptosis of MCAO rats was largely increased by OE‐TIMP1 (*p* < .01). The Western blot assay further confirmed that exercise weakened the apoptosis of the cerebral cortex tissues for MCAO rats. This was evidenced by a notable increase of B‐cell lymphoma‐2 (Bcl‐2) expression, and evident decreases of BCL2‐Associated X (Bax) and caspase 3 expressions in the exercise group rats, while the effects were partially reversed after overexpressing TIMP1 (Figure 6, *p* < .01).

### 3.5 OE‐TIMP1 partially blunted the salutary effects of exercise on MCAO‐induced oxidative stress damage

**Figure 7:**
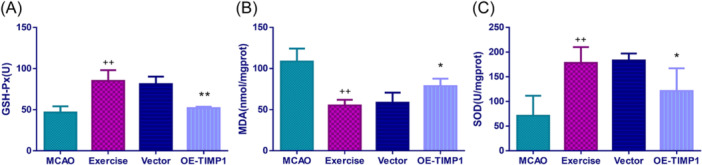
Exercise upregulated GSH‐PX and SOD contents but downregulated MDA content in MCAO rats by inhibiting TIMP1. After MCAO, the contents of GSH‐Px (A), SOD (B) and MDA (C) in the cerebral cortex tissues of MCAO rats were measured using chemical colorimetry. ^++^ *p* < .01 versus MCAO; *p < .05, **p < .01 versus Vector. Data were analyzed using One‐way ANOVA with Turkey tests. *n* = 6. GSH‐Px, glutathione peroxidase; SOD, superoxide dismutase; MDA, malondialdehyde.

This study also found that exercise apparently reduced MCAO‐triggered oxidative stress damage in the cerebral cortex tissues, as evidenced by decreased MDA production, and upregulated GSH‐PX and SOD production (Figure 7, *p* < .01). Nevertheless, OE‐TIMP1 weakened the elevations of GSH‐PX and SOD but enhanced the activities of MDA in MCAO rats in the presence of exercise (Figure 7, *p* < .01).

### 3.6 Exercise inactivated HIF‐1α/vascular endothelial growth factor (VEGF) pathway in MCAO rats by inhibiting TIMP1

**Figure 8:**
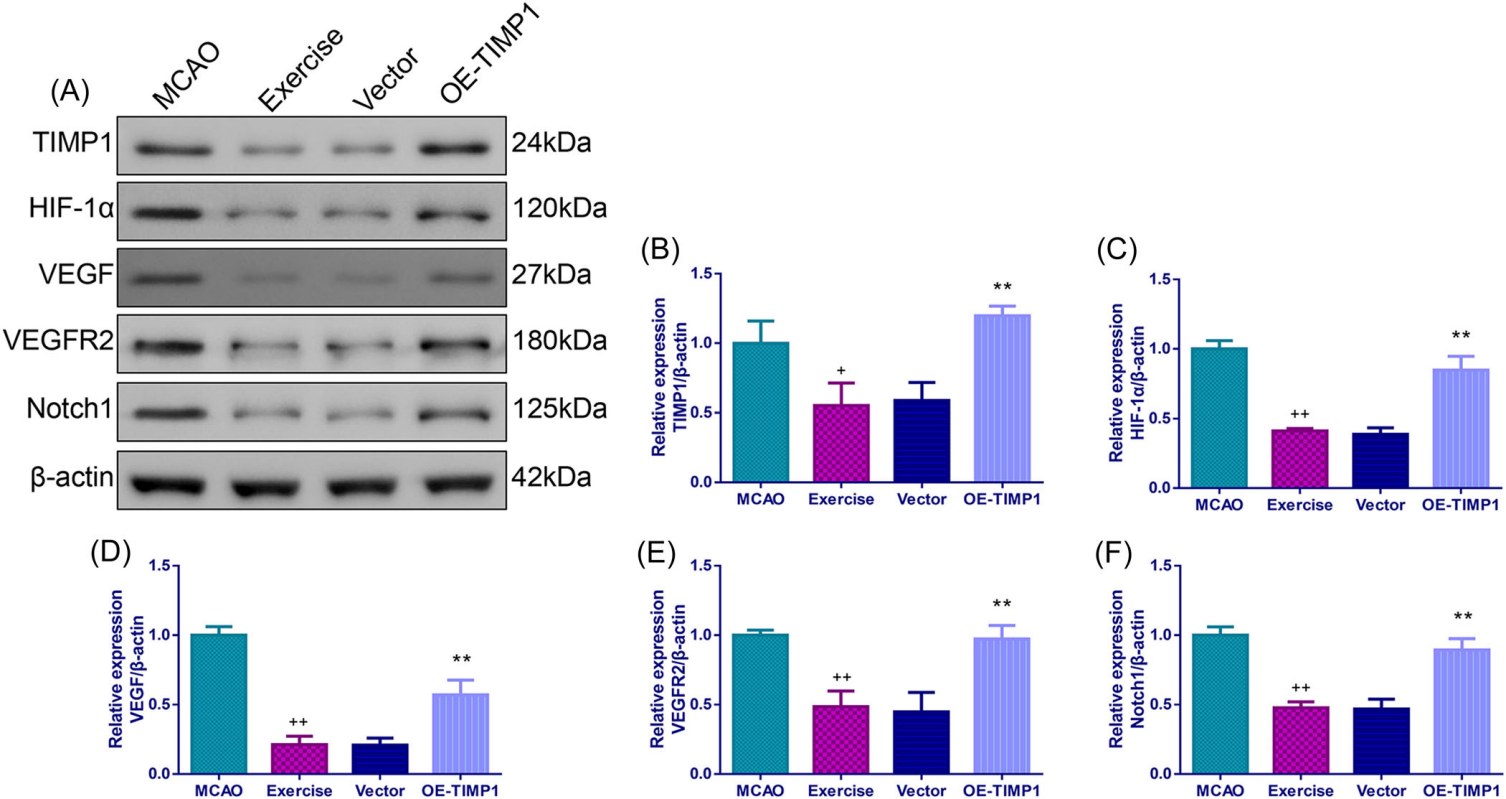
Exercise inactivated HIF‐1α/VEGF pathway in MCAO rats by inhibiting TIMP1. After MCAO, Western blot assay was employed to measure TIMP1, HIF‐α, VEGF, VEGFR2, and Notch1 protein expressions in rat cerebral cortex. (A) Representative images for Western blot assay of TIMP1, HIF‐α, VEGF, VEGFR2, and Notch1 levels in the cerebral cortex. Quantifications of TIMP1 (B), HIF‐α (C), VEGF (D), VEGFR2 (E), and Notch1 (F) levels in the cerebral cortex. ^+^ *p* < .05, ^++^ *p* < .01 versus MCAO; **p < .01 versus Data were analyzed using One‐way ANOVA with Turkey tests. *n* = 3. Vector. Note: HIF‐α, hypoxia‐inducible factor‐alpha; VEGF, vascular endothelial growth factor; VEGFR2, vascular endothelial growth factor receptor 2, Notch1, neurogenic locus notch homolog protein 1.

The data in Figure 8 implied that TIMP1, HIF‐α, VEGF, vascular endothelial growth factor receptor 2 (VEGFR2), and neurogenic locus notch homolog protein 1 (Notch 1) protein expressions in the exercise group were lower than those in the MCAO group (*p* < .05). More important, OE‐TIMP1 partially reversed these situations (Figure 8, *p* < .01).

## 4 DISCUSSION

It has been reported that neurological dysfunction is frequently observed in patients with cerebral IS.^23^ Additionally, cerebral IS also leads to the emergence of neuron death and cerebral infarction.^35^ Therefore, evaluating neurological dysfunction, neuronal death, and cerebral infarction size is important to judge the degree of brain injury after stroke. In addition, although the implementation of post‐stroke prevention strategies can reduce the recurrence rate, it is still not ideal in some areas. Compared with the treatment after stroke, prevention is a better choice, such as ischemic preconditioning, exercise preconditioning, electroacupuncture preconditioning, etc., which can activate various biomolecules to induce endogenous tolerance of ischemia and IRI.^36^, ^37^ The tolerance mechanisms generated by nonfatal ischemia and other causes in body tissues can provide strong protection.^38^ Exercise preconditioning is easy to popularize and study because it is easy to implement and less restrictive. Exercise preconditioning can also enhance ischemia tolerance, protect the cardiovascular system, and play a role in promoting neurovascular regeneration and inducing protective factors.^39^, ^40^ The results of this experiment showed that MCAO rats with exercise preconditioning had lower neurological deficit scores, neuronal death rates, and cerebral infarction area, which were consistent with Pan et al.'s study of treadmill exercise on cerebral ischaemic injury, treadmill exercise improved neurobehavioral scores, cerebral infarction, and neuronal loss.^41^

It was found that TIMPs should be taken into account to realize the neuroprotective mechanisms after CIRI. Matrix metalloproteinases (MMPs) control angiogenesis by exerting a crucial role in degrading the basement membrane and extracellular matrix.^42^ However, TIMP1 is a specific endogenous inhibitor of MMPs. Upon acute cerebral ischemia, TIMP1 expression was obviously upregulated to be involved in neurodegeneration.^43^ The use of TIMPs may be an effective way to prevent and treat CIRI. Therefore, the decreased expression of TIMP1 manifests a crucial role in the neuroprotective process after CIRI. A study has reported that exercise improves cardiac function by regulating the concentrations of MMP‐9 and TIMP1 in diabetic cardiomyopathy mice.^19^ Another study has shown that early exercise obviously weakens the ischemia‐mediated upregulation of TIMP1, thereby ameliorating ischemic brain damage.^44^ Also, this research has found that TIMP1 is highly expressed in the cerebral cortex of MCAO rats and exercise preconditioning weakens TIMP1 expression. More interestingly, the reduced effects of exercise preconditioning on neurological deficit scores, neuronal death rate, and cerebral infarction area in MCAO rats were partially reversed by TIMP1 overexpression.

A published study has indicated that oxidative stress injury is important in secondary brain damage triggered by reperfusion.^45^ MDA is the product of lipid peroxide; SOD is the main defense line to prevent tissue damage caused by reactive oxygen species; GSH is an endogenous antioxidant that can react directly with reactive oxygen species.^46^ One report has proposed that pre‐ischemic exercise training can elevate the activity of SOD but reduce the content of MDA in MCAO rats.^47^ In line with the above results, our study also discovered that exercise apparently restrained MCAO‐triggered oxidative stress damage of the cerebral cortex tissues, concomitant with decreased MDA production, and upregulated GSH‐PX and SOD productions. More interestingly, TIMP1 overexpression partially blunted the salutary effects of exercise on MCAO‐induced oxidative stress damage, unveiling that the protective effects of exercise on CIRI might be linked to the antioxidant stress of MCAO rats.

A previous study has revealed that apoptosis may exhibit a pivotal role in the pathological process of CIRI.^48^ Apoptosis is associated with the expression of Bcl‐2 (inhibition of apoptosis) and Bax (promotion of apoptosis). The elevated expression of Bax leads to an increase in mitochondrial permeability, which in turn triggers the release of cytochrome c to the cytoplasm and the activation of caspase 3, thereby promoting apoptosis.^49^ It has been reported that exercise can prevent lesions caused by ischemia in the CA1 region by reducing apoptosis.^50^ Our study also discovered that exercise weakened apoptosis, with notable increases of Bcl‐2 protein expression, and evident decreases of Bax and caspase 3 protein expressions in MCAO rats, while these effects were partially reversed after overexpressing TIMP1.

Mounting evidence has revealed that the HIF‐VEGF‐Notch pathway participates in the pathogenesis of IS, and modulating the HIF/VEGF/Notch pathway will change IS prognosis.^51^, ^52^ VEGF, as a cytokine that promotes the growth of vascular endothelial cells, increases capillary osmotic pressure, and stimulates angiogenesis, thereby playing the role of nutritional nerve and neuroprotection.^53^ HIF‐1α is an important nuclear transcription factor in mammals under hypoxia conditions.^54^ It has been found that ligustilide can reduce blood‐brain barrier permeability in cell models stimulated by oxygen‐glucose deprivation via the HIF‐1α/VEGF pathway and Aquaporin‐4.^55^ Under extreme conditions of cerebral ischemia‐reperfusion, HIF‐1α is pathologically overexpressed in tissue cells, resulting in abnormal elevations of VEGF and Notch1, which may not be conducive to the protection of neural function.^56^ A published study has suggested that exercise preconditioning can protect ischemic brain tissues by regulating the expressions of HIF‐1α and VEGF, thereby alleviating ischemic injury.^57^ Consistent with the published study, the present study revealed that exercise prominently repressed TIMP1, HIF‐α, VEGF, VEGFR2, as well as Notch 1 protein expressions in MCAO rats. More importantly, the suppression of exercise on the HIF‐VEGF‐Notch pathway in MCAO rats was partially reversed by TIMP1 overexpression, illustrating that the mechanisms of exercise to prevent CIRI may be associated with the HIF‐VEGF‐Notch pathway.

Our study revealed that exercise preconditioning suppressed the HIF‐VEGF‐Notch pathway through TIMP1, enhanced the body's antioxidant stress and antiapoptotic capacity, and exerted neuroprotective effects on CIRI, thereby providing a basis for the prevention of stroke in high‐risk populations.

## 5 LIMITATIONS

However, there were some limitations in the study. One major limitation was that we didn't exclude the effects of electroshock on the development of CIRI, despite 1.5 mA electroshock may be safe for rats. In addition, we didn't test inflammation factor levels of the cerebral cortex tissues in the study. In the future, we will increase an electroshock group and treat the rats with electroshock. Moreover, we will detect the inflammation factor levels of the cerebral cortex tissues, thus making the key findings of the study more convincing.

## AUTHOR CONTRIBUTIONS

**Xiangbo Meng**: Conceptualization; Data curation; Formal analysis. **Hui Yang**: Investigation; Methodology; Resources. **Feifeng Chen**: Investigation; Supervision. **Baohua Li**: Software; Validation. **Yan Wu**: Formal analysis; Visualization. **Rong Wang**: Formal analysis; Writing—review and editing.

## CONFLICT OF INTEREST STATEMENT

The authors declare no conflicts of interest.

## ETHICS STATEMENT

All procedures involving animals adhered to the National Institutes of Health Guide for the Care and Use of Laboratory Animals and followed the ARRIVE checklist. Ethics approval was acquired from the Ethics Committee of Zhejiang Eyong Pharmaceutical Research and Development Center (SYXK (Zhe) 2021‐0033).

## Supporting information

Supporting information.

## Data Availability

The datasets generated during and/or analysed during the current study are available from the corresponding author upon reasonable request.
